# Ex vivo isolated human vessel perfusion system for the design and assessment of nanomedicines targeted to the endothelium

**DOI:** 10.1002/btm2.10154

**Published:** 2020-01-28

**Authors:** Taras Lysyy, Laura G. Bracaglia, Lingfeng Qin, Claire Albert, Jordan S. Pober, George Tellides, W. Mark Saltzman, Gregory T. Tietjen

**Affiliations:** ^1^ Department of Surgery Yale School of Medicine New Haven Connecticut; ^2^ Department of Biomedical Engineering Yale University New Haven Connecticut; ^3^ Department of Chemical Engineering Yale University New Haven Connecticut; ^4^ Department of Immunobiology Yale School of Medicine New Haven Connecticut

**Keywords:** clinical translation, drug delivery systems, endothelial cell targeting, ex vivo perfusion, molecularly targeted therapies, nanoparticle accumulation, nanotechnology

## Abstract

Endothelial cells play a central role in the process of inflammation. Their biologic relevance, as well as their accessibility to IV injected therapeutics, make them a strong candidate for treatment with molecularly‐targeted nanomedicines. Typically, the properties of targeted nanomedicines are first optimized in vitro in cell culture and then in vivo in rodent models. While cultured cells are readily available for study, results obtained from isolated cells can lack relevance to more complex in vivo environments. On the other hand, the quantitative assays needed to determine the impact of nanoparticle design on targeting efficacy are difficult to perform in animal models. Moreover, results from animal models often translate poorly to human systems. To address the need for an improved testing platform, we developed an isolated vessel perfusion system to enable dynamic and quantitative study of vascular‐targeted nanomedicines in readily obtainable human vessels isolated from umbilical cords or placenta. We show that this platform technology enables the evaluation of parameters that are critical to targeting efficacy (including flow rate, selection of targeting molecule, and temperature). Furthermore, biologic replicates can be easily produced by evaluating multiple vessel segments from the same human donor in independent, modular chambers. The chambers can also be adapted to house vessels of a variety of sizes, allowing for the subsequent study of vessel segments in vivo following transplantation into immunodeficient mice. We believe this perfusion system can help to address long‐standing issues in endothelial targeted nanomedicines and thereby enable more effective clinical translation.

## 1 INTRODUCTION

Molecularly‐targeted nanoparticles (NPs) have the potential to be powerful tools for site‐specific drug delivery to treat many pathologies.^1^, ^2^, ^3^ However, despite numerous innovations and applications explored in recent years, clinical impact has yet to be realized.^4^, ^5^, ^6^ Even in cases with promising preclinical results in rodents, subsequent clinical trials have shown only modest efficacy in humans.^7^ This lack of clinical relevance has led some to question whether targeted nanomedicines are a viable therapeutic technology.^8^ Notably, the majority of this prior research has attempted to target extra‐vascular cells (e.g., in solid tumor) via intravascular NP delivery. In contrast to poorly accessible extra‐vascular cells, vascular endothelial cells (ECs) are directly accessible to circulating NPs. Indeed, extensive research on EC‐targeted NPs has demonstrated that molecular‐targeting can dramatically enhance the retention of NPs on ECs both in vitro and in vivo.^9^, ^10^, ^11^ Furthermore, ECs play a critical role in homeostasis and inflammation, and therefore represent an attractive cell population for treatment with targeted nanomedicines in a variety of disease indications.^12^

The predominant path for preclinical development of EC‐targeted NPs typically begins with static cell culture models.^13^, ^14^, ^15^ These in vitro models are easy to use, available to most laboratories, and produce clear quantitative readouts to assess targeting efficacy (i.e., mean fluorescence intensity of cells treated with NPs encapsulating fluorescent dye). However, in most cases, cells in culture have significantly different biological properties compared to cells in their native tissue environment. Moreover, the physiologic and environmental characteristics of static cell culture systems are a poor proxy of a complex in vivo system. Consequently, it is not surprising that NP targeting in vitro is typically poorly predictive of efficacy in vivo.^4^, ^16^ Microfluidic flow chambers may provide some improvement by enabling study of NP targeting to EC under physiologic shear conditions, which has been shown to effect NP internalization.^17^, ^18^ However, such microfluidic systems may not be available to all researchers and still do not replicate the native 3D archictecture and surrounding cell types of native vessel beds.

Animal models, in contrast to cells in culture, provide a more complex physiological setting within which to evaluate NP targeting efficacy.^19^, ^20^, ^21^ Notably, effective vascular targeting of NPs in mouse models has been achieved in select tissues (e.g., lung and brain).^9^, ^22^, ^23^, ^24^, ^25^, ^26^, ^27^, ^28^ These studies have revealed the critical importance of molecular properties on NP targeting efficacy. They have further highlighted the need to optimize both target‐receptor selection and NP‐ligand properties to ensure effective delivery. Engineering such systems depends on specific characteristics of the receptor targets displayed on the luminal surface of the ECs and the binding properties of the targeting ligand conjugated to the NP surface.^29^ As a consequence, it is unlikely that the same reagents that bind to ECs of mice will be relevant in humans. Moreover, genetically identical mice lack the natural anatomic and physiologic variability associated with the human population.^19^ Finally, experiments conducted in a complex animal model do not allow for isolation or control over key variables (i.e., perfusate composition, temperature, flow rate, and circulation half‐life) obscuring the influence of NP design and environmental factors on NP targeting efficacy.

The aforementioned challenges associated with simple cell culture models and complex animal models suggest the need for approaches that can enable evaluation of targeted NP delivery in three‐dimensional (3D) human tissues under circulation. Recently, we have investigated targeted NP delivery to human renal vasculature during ex vivo normothermic machine perfusion (an emerging clinical modality for improving the condition of solid organs prior to transplantation^30^). This study allowed us to measure the targeting of anti‐CD31 NPs to ECs within native vascular beds of human kidney.^31^ Though ex vivo perfusion of human organs provides a unique opportunity to evaluate targeted NPs in a native setting, access to nontransplanted human organs is limited and the size of the experiments (typically 500 ml perfusion volume in a human kidney) makes these experiments highly costly. Additionally, it is not currently possible to study long term drug effects in these human tissues.

In other recent work, we have circumvented these issues by delivering NPs to small diameter human vessels ex vivo. These small vessels have the potential for long term evaluation following transplant in humanized mice. In one such study, we have treated human coronary artery segments ex vivo with siRNA‐loaded NPs that accumulated within ECs during the treatment period.^32^ After NP treatment, the human arteries were transplanted as interposition grafts into the infrarenal abdominal aortae of immunodeficient mice. Over the 14 day study, NPs provided a prolonged knock‐down of major histocompatibility complex (MHC) Class II molecules, which reduced inflammatory‐stimulated damage by adoptively transferred human peripheral blood mononuclear cells from a donor allogeneic to the artery graft.^32^ These quantitative studies of NP interactions with human tissue could be optimized by a testing platform that allows for controlled perfusion of multiple vessels collected from the same donor.

Here we have developed a testing platform that enables us to use readily obtained human vessel segments in isolation ex vivo. In this platform—which we call the IVPS (isolated vessel perfusion system)—vessel segments are maintained as 3D structures with an intact native endothelial lining, allowing quantification of NP accumulation and cellular response. In proof of principle studies, we used this system to compare the effect of temperature, flow rate, and cellular target on NP targeting efficacy to vascular ECs under flow. Furthermore, we used the system to deliver NPs to vessels ex vivo, and then evaluated the persistence of NPs after in vivo implantation of the treated vessels in immunodeficient mice. In sum, we have developed a reliable, simple, and inexpensive testing platform for quantitative assessment of vascular‐targeted nanomedicines in readily accessible human tissues. We believe this platform can complement existing models for NP design optimization (cell culture, in vivo animal models, ex vivo nontransplanted human organs) and thereby improve clinical translational of vascular‐targeted nanomedicines.

## 2 RESULTS

### 2.1 Fresh collection of umbilical arteries from C‐section ensures endothelial integrity

**Figure 1:**
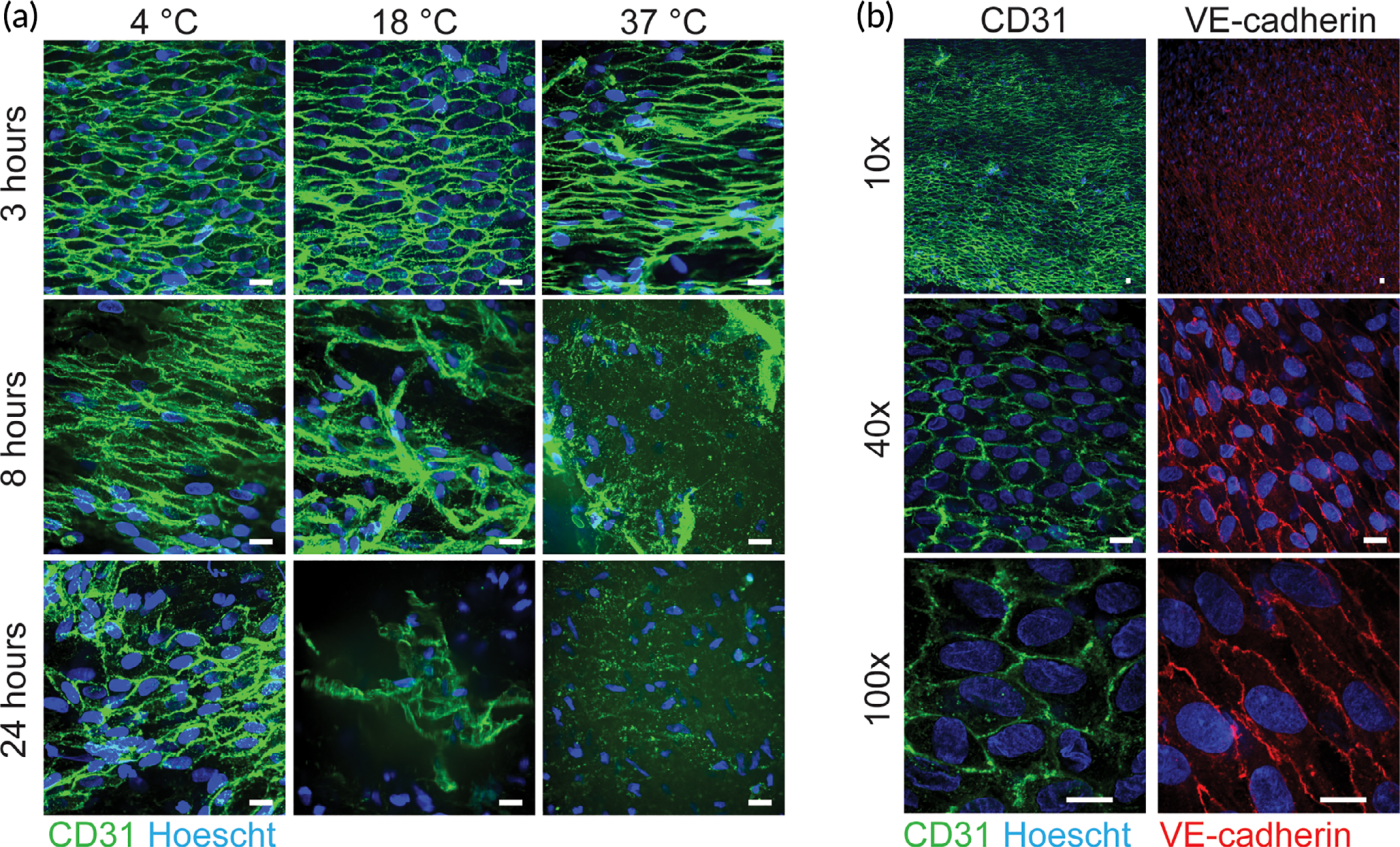
Fresh Collection of Umbilical Arteries from C‐Section Ensure Endothelial Integrity. (a) Sections obtained from a single umbilical artery were stored at the noted temperature and stained with fluorescent CD31 (green) to visualize endothelium over time. (b) Sections from a single umbilical artery show endothelial cell coverage and cellular junctions at increasing magnification (10x, 40x, and 100x). Vessels are stained for CD31 (green) and VE‐cadherin (red) at 3 hours after C‐section; vessels were stored at 4˚C prior to staining. All scale bars are 20 μm

Human umbilical arteries can potentially provide an abundant, accessible, and consistent source of intact endothelium for NP targeting efficacy experiments. We first sought to identify optimal conditions to maintain endothelial integrity in these arteries prior to experimentation. Confocal microscopy of *en face* mounted specimens revealed that the endothelial layer of umbilical arteries was continuous and intact when evaluated within 3 hrs after cesarean section (C‐section) for tissues stored at 4°C (Figure 1(a)); 3 hrs was the earliest time point we could reliably retrieve arteries from the delivery room and then dissect for experimentation. Prolonged tissue storage of 8 or 24 hrs at 4°C reduced EC layer integrity. Umbilical cords maintained at either 18°C (room temperature) or 37°C after C‐section also appeared to have intact EC layers at the 3 hr time point, but similar to 4°C, EC were lost after prolonged storage. Based on this evidence, we concluded that tissues should be obtained fresh from C‐section (as opposed to unscheduled, natural birth) so that the time and handling conditions between tissue collection and experiment could be reliably controlled. For all subsequent experiments, we maintained cords at 4°C after recovery to reduce cellular metabolism and any injury associated with warm ischemia.

Finally, we assessed the integrity of EC junctions following recovery and storage at 4°C for 3 hrs prior to staining. Vessels were fixed, permeabilized, and stained for either CD31 or VE‐cadherin as an indicator of EC coverage and junctional integrity (Figure 1b). Confocal images at a variety of magnifications (×10, ×40, and ×100) demonstrated intact EC layers with well‐defined EC junctions. Collectively, these data suggest that umbilical arteries recovered from C‐section can provide a reliable source of human vessels for subsequent ex vivo experimentation.

### 2.2 The IVPS enables ex vivo perfusion of human vessel segments

**Figure 2:**
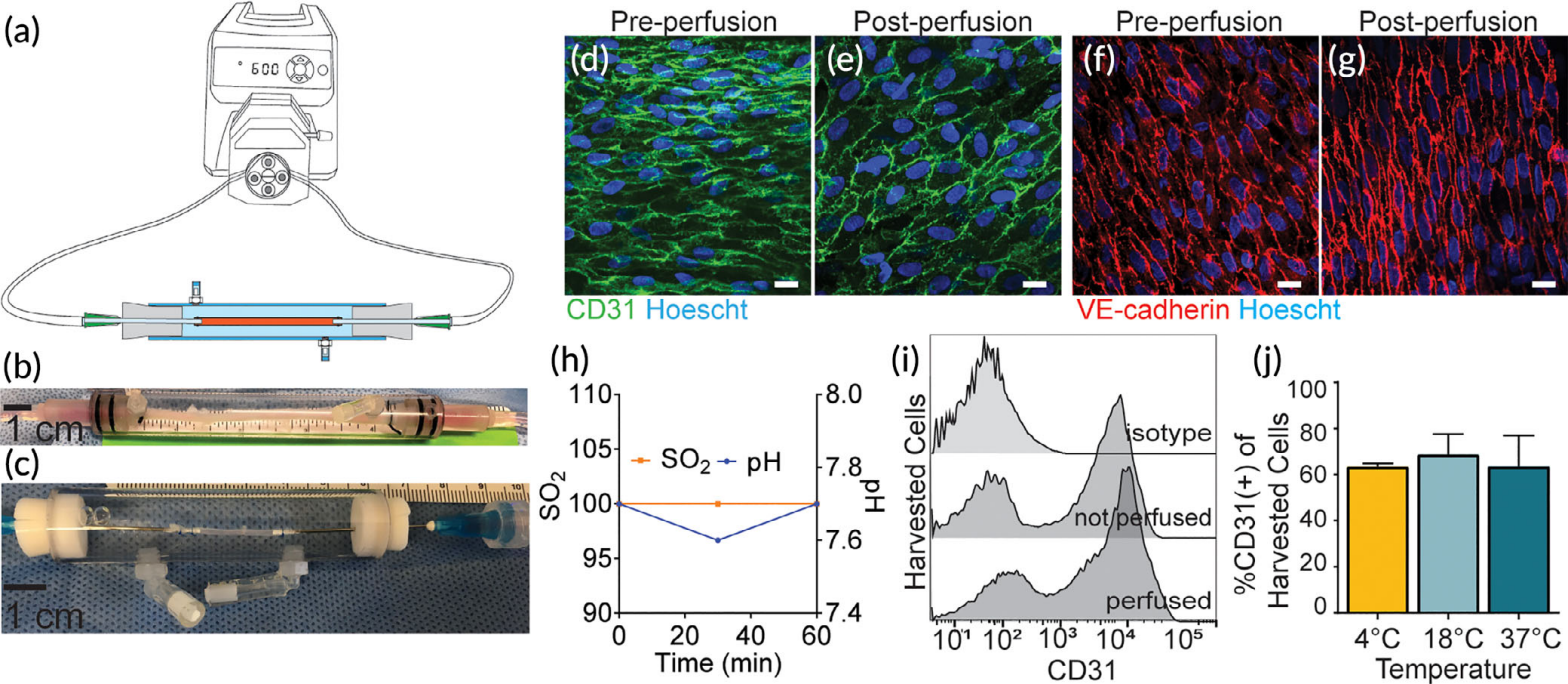
The IVPS Enables ex vivo Perfusion of Human Vessel Segments. (a) Schematic illustrating the perfusion loop construction with an ex vivo vessel perfusion chamber attached. (b) Photographs of an umbilical artery and (c) placenta artery secured in perfusion chambers with 1 cm scale bar. Representative confocal images, stained for CD31 (green) and VE‐cadherin (red) of vessel segments from unperfused vessel (d & f), and vessels perfused at 2.5 mL/min at 37˚C (e & g). Scale bars are 20 μm. (h) pH and oxygen saturation in the perfusate throughout a 1‐hour perfusion show levels remain stable. (i) Representative histogram of cells harvested from a perfused and unperfused vessel shows that a distinct population of CD31+ cells can be distinguished from the harvested cell population. (j) Bar graph depicting the percent of CD31+ endothelial cells harvested following perfusion as a function temperature

To develop a platform for assessment of targeted NP interactions with human blood vessels, we constructed a closed‐loop, ex vivo perfusion system. A schematic of our design is shown in Figure 2a. In this system, a peristaltic pump drives fluid at a controllable volumetric flow rate. By adjusting pump speed and tubing resistance, the IVPS can be adjusted to control flow rate, intravascular pressure, and shear stress at the surface of the vascular lumen (Table 1). The modular design allows for human vessel segments of various diameters to be connected to the flow loop by first cannulating with a gavage needle of matching diameter on both sides of the vascular graft, and then attaching the needles to the tubing of the loop. We have also included a custom‐designed chamber sealed with rubber stoppers around the cannulated needles to enclose the vessels and provide an aqueous extra‐vascular environment (Figure 2b,c). Multiple isolated vessel setups (up to 8 in parallel) can be simultaneously perfused to produce biological replicates for side‐by‐side testing under identical conditions.

**Table 1:** Typical shear stress and pressure during perfusion

Vessel inner diameter	Volumetric flow rates (ml/min)	Calculated shear stress (dyne/cm^2^)	Mean intravascular pressure (mmHg)
1.9–2.4 mm (human umbilical artery)	1.5	0.3–2.0	21.6
	2.5	0.4–3.3	21.2
	5	0.5–6.6	22.8

To validate the utility of the IVPS, we first assessed the maintenance of intact, healthy endothelium during perfusion. Human umbilical arteries were dissected from a single umbilical cord and mounted in the IVPS. Multiple vessel segments of identical length were prepared and tested in parallel. *En face* confocal imaging demonstrated good integrity of the EC layer and junctions before and after perfusion at 37°C and 2.5 ml/min for 1 hr (Figure 2d–g). Perfusate pH and saturated oxygen levels were also measured as an indicator of physiologic stability over the course of a typical NP targeting experiment; these parameters remained stable over this time frame (Figure 2h). After the 1‐hr perfusion, a population of CD31+ ECs were identified from a mixed population of cells harvested by collagenase treatment. The recovered CD31+ population was consistent when collected from vessels either before or after perfusion (Figure 2i). Finally, vessels from the same donor were perfused simultaneously at either 4, 18, or 37°C as a demonstrations of the capacity of the system to simultaneously evaluate different perfusion conditions. Again, a consistent proportion of ECs were retrieved from each vessel segment (Figure 2j).

### 2.3 Physical perfusion parameters affect NP accumulation on endothelium

**Figure 3:**
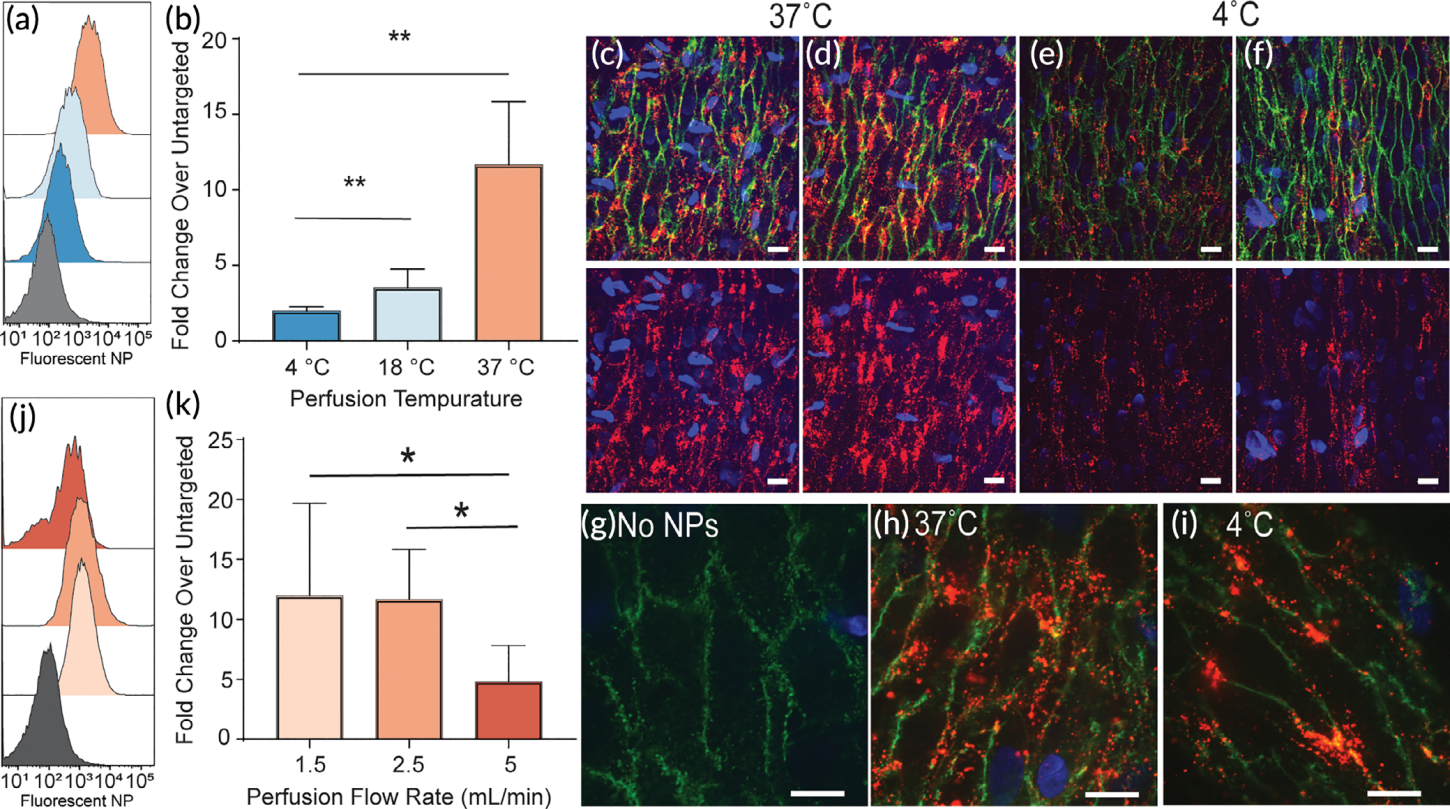
Physical perfusion parameters affect NPs accumulation on endothelium. (a) Representative histograms showing the relative magnitude of fluorescent Ulex‐targeted NP accumulation on isolated endothelial cells at different perfusion temperatures. (b) Summary data from triplicate perfusion experiments are shown normalized to vessels perfused with untargeted NPs at 37˚C. **P <0.01 using ANOVA and a post hoc Tukey's Test. En face confocal images of vessel segments perfused at 37˚C (c,d,h) or 4 ˚C (e,f,i). Vessels are stained with antiCD31‐FITC (green) and Hoechst nuclear stain (blue); NP are depicted in red. Bottom row of (c‐e) shows NP and nuclear stain for corresponding images in top row. (g) Representative image of control vessel without NP perfusion. Scale bars are 20 μm. (j) Representative histograms showing the relative magnitude of fluorescent NP accumulation on isolated endothelial cells at different flow rates. (k) Summary data from triplicate perfusion experiments are shown normalized to cells from vessels perfused with untargeted NPs at 2.5 mL/min. *P <0.05 using ANOVA and a post hoc Tukey's Test

Temperature is a critical aspect of optimal organ preservation during ex vivo perfusion for transplant; both hypothermic and normothermic perfusion conditions are under clinical evaluation.^33^ Thus, we next evaluated the effect of perfusion temperature on targeted NP retention on ECs. PLA–PEG NPs conjugated to the Ulex europaeus agglutinin I (Ulex) protein were delivered to human umbilical arteries under perfusion at 4, 18 (room temperature), or 37°C for 60 min (Figure 3a–f). This lectin binds to human blood group H, whose expression is shared by human erythrocytes and ECs and is often used as an EC‐specific marker in tissues. In comparison to nontargeted NPs perfused at 37°C (shown in gray in Figure 3a), there was higher association of targeted NPs at all temperatures tested. However, the magnitude of the enhanced retention demonstrated a clear temperature dependence; 4°C (~1.4 fold), 18°C (~3 fold), and 37°C (~10 fold). This result was confirmed by confocal *en face* imaging of the intact vessels (Figure 3c‐i); more NPs were visible after perfusion at 37°C (Figure 3c,d,h) than at 4°C (Figure 3e,f,i). Notably, the spatial pattern of NP appears to be primarily junctional at 4°C and less restricted to the EC junctions at 37°C. Additional ~2 hr room temperature incubation after perfusion at 37°C led to even more spatial shift of NP away from the junctions perhaps indicating NP internalization (Figure S1). While a detailed study of NP internalization is beyond the scope of this method‐focused manuscript, these data suggest that the IVPS could be used to study the effects of temperature on NP internalization in intact vessels.

The flow rate of perfusion was also assessed for effects of NP targeting to ECs. NPs were delivered at flow rates of 1.5, 2.5, or 5 ml/min for 60 min. The lower flow rates demonstrated similar levels of NP targeting (Figure 3j,k). Reduced levels of NP were found on ECs perfused at 5 ml/min. Again, this result was confirmed by fluorescence imaging of intact vessels after perfusion, where more NP accumulation is observed at 1.5 and 2.5 ml/min perfusion compared to 5 ml/min (Figure S2). While reduced NP retention was observed at 5 ml/min, we also noted reduced EC recovery and potential areas of sheared endothelium at this flow rate (Figure S2). It is possible that 5 ml/min may reflect the upper limit of experimental parameters for assessing NP binding in this system. Nevertheless, we conclude that perfusion at 37°C between 1.5 and 2.5 mL/min results in indistinguishable levels of NP targeting efficacy to umbilical arteries. It should be noted, however, that these experiments used cords from three separate donors with natural variability in vessel diameter (typically 1.9–2.4 mm diameter) that translates to corresponding variability in shear (Table 1). This variation in shear may account for the relatively large observed error bars that could be obscuring the influence of fluid dynamics on NP targeting efficacy.

### 2.4 Molecular target selection can enhance targeted‐NP accumulation

**Figure 4:**
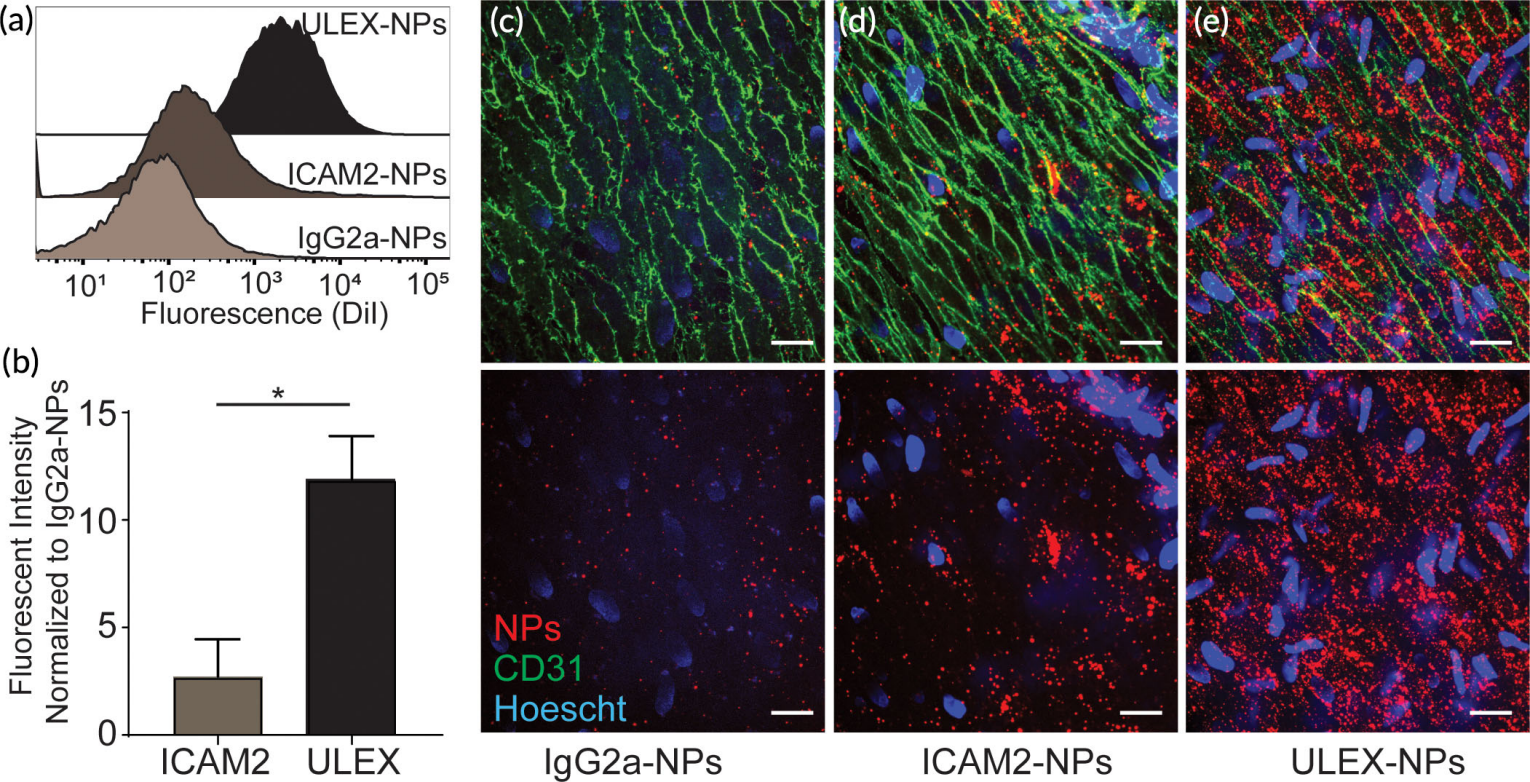
Molecular Target Selection Can Enhance Targeted‐NP Accumulation (a) Representative histograms show relative levels of NP accumulation for vessels perfused with either ULEX‐targeted NPs or ICAM‐2‐targeted NPs. (b) Summary of triplicate data normalized to the fluorescent intensity of cells from vessels perfused with isotype‐targeted NPs. *P <0.001 using un unpaired T Test. Representative en face confocal images for vessel segments perfused with IgG‐NPs (c), ICAM‐2‐NPs (d) and ULEX‐NPs (e). AntiCD31‐FITC is shown in green, Hoescht nuclear stain in blue, and NPs in red. Scale bars are 20 µm

To assess the effect of ligand/target‐receptor selection, NPs conjugated to either antihuman endothelial intercellular adhesion molecule 2 (ICAM‐2) antibodies or to Ulex lectin were introduced by perfusion of umbilical arteries. ICAM‐2 (CD102), like blood group H, is highly and selectively expressed on human ECs. After 60 minutes, ECs were harvested, and the NPs retained on cells were quantified. ICAM‐2‐targeted NPs were found on cells at three‐fold higher levels compared to isotype control NPs. Ulex‐targeted NPs exhibited significantly stronger binding that ICAM‐2 at 11‐fold compared to the isotype control NPs (Figure 4a,b). This result was again confirmed with qualitative fluorescence imaging showing more Ulex‐targeted NPs on the endothelial layer than ICAM‐2‐targeted NPs (Figure 4c‐e). These data demonstrate the impact of ligand/target‐receptor selection on NP‐targeting efficacy.

### 2.5 Perfused vascular grafts retain human endothelium and ICAM2‐NPs at 7 days post‐transplant

**Figure 5:**
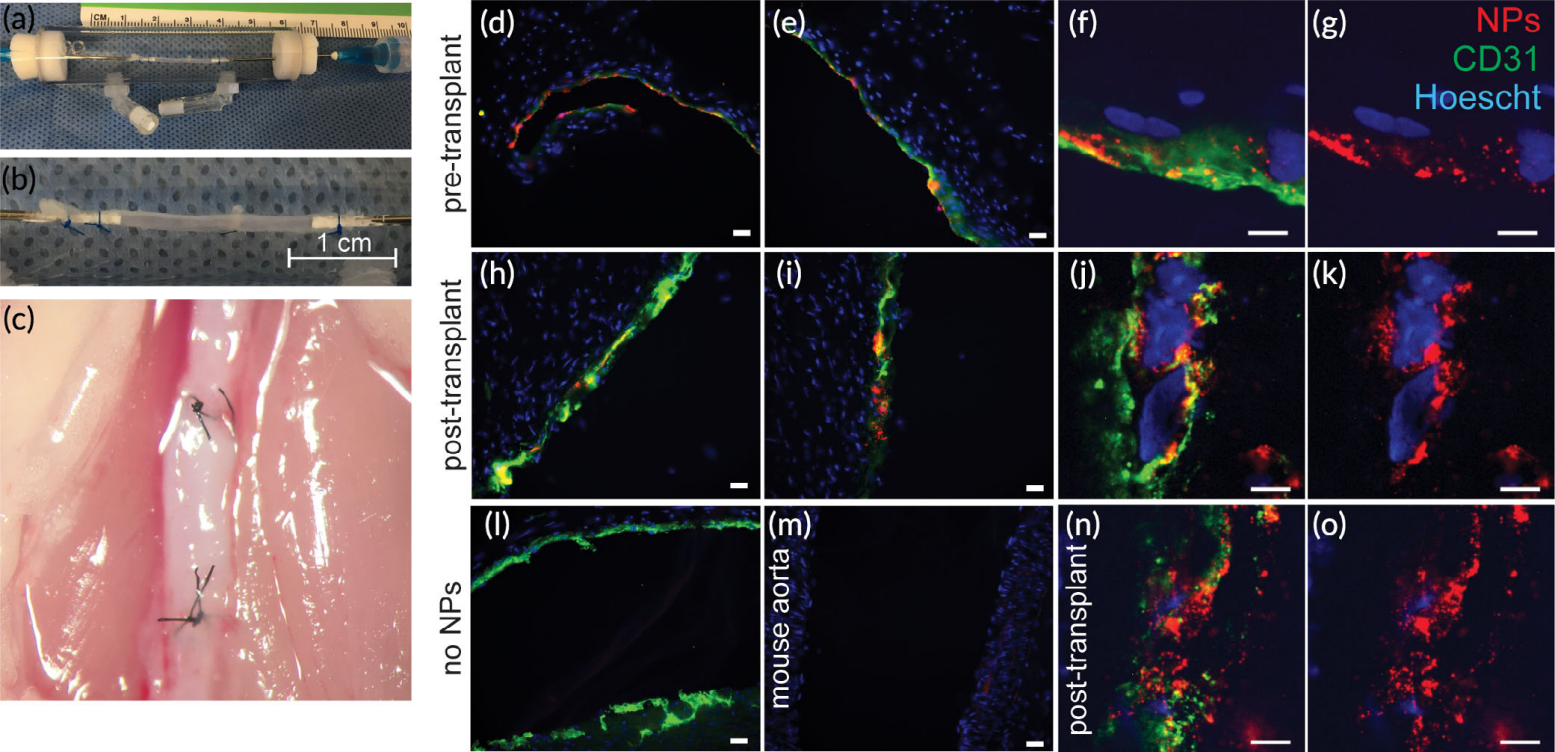
Perfused Vascular Grafts Retain Human Endothelium and ICAM2‐NPs 7 Days Post‐Transplant. Depiction of human placenta arteries mounted into a perfusion chamber (a) and (b), prior to perfusion with ICAM‐2‐targeted NPs or Isotype Control NPs as a control. Vascular grafts were implanted in the infrarenal aorta of SCID/beige mice (c) and then harvested 7 days later. Frozen sections of these grafts, stained with anti CD31‐FITC and Hoescht are shown prior to transplant (d‐g), and 7 days after transplant (h‐k & n,o). (l) shows a section of placenta artery that was not perfused with NPs, and (m) shows a section of mouse aorta that negative for human CD31. Scale bars are 20 μm

To assess the long‐term durability of tissues perfused in our system, as well as the persistence of NP association with the vessel tissues after perfusion, the IVPS can be formatted for the perfusion of even smaller diameter vessels that are suitable for implantation in mice. To this end, human placenta arteries were dissected and perfused with ICAM‐2‐targeted NPs in the IVPS. Perfused vessels were flushed of any unbound NPs and subsequently implanted into the mouse arterial circulation at the infrarenal aorta (Figure 5a–c). Fluorescence images of vessels harvested 7 days after transplant reveal that the human endothelial layer is preserved; a continuous layer was seen in both the pretransplant (Figure 5d–g) and post‐transplant (Figure 5h–k,n,o) images. Furthermore, fluorescent NPs are visible immediately after the perfusion (pretransplant; Figure 5d–g) as well as 7 days post‐transplant (Figure 5h–k,n,o). The continued presence of NPs for 1 week in transplanted vessel segments demonstrates the capability of the system to allow association of NPs under controlled conditions for subsequent study of their effects after transplantation.

## 3 DISCUSSION

The IVPS described here provides a simple experimental platform to test EC targeted nanomedicines in a human‐tissue environment. Alternative platforms to investigate vascular‐targeted NP designs are most often in cell culture, animal models, and more recently ex vivo human organ perfusion. We believe that this platform, which allows for experimentation on vascular segments with an intact and appropriately organized human endothelial lining, complements these existing experimental platforms in four key ways.

First, similar to human organ perfusion, the IVPS provides a binding environment relevant to delivery in human tissues. Isolated human vessels under flow exhibit appropriate binding challenges and physical parameters that are not well represented in cell culture. Some of these parameters could be achieved in a mouse or other small animal model (competition for binding, fluid dynamics), but experiments in the IVPS can be conducted with human tissues. Furthermore, the ex vivo platform allows for the controlled manipulation of environmental parameters (temperature and intravascular flow rate, pressure and shear stress), which could be important variables that reveal the underlying biophysics of NP delivery. Perfusate composition is also a critical variable that can be systemically explored with the IVPS. Here, we have conducted the tests using a standard EC culture media (M199 with 20% FBS), but it is also possible to evaluate NP delivery within other perfusates relevant to ex vivo perfusion (e.g., whole blood, washed red blood cells, or cell‐free preservation fluids like University of Wisconson's solution). The inclusion of washed red blood cells, for example, would allow for assessment of competitive binding between the red blood cells and ECs.

Second, the IVPS is a small volume system designed to enable rapid optimization of nanomedicine formulations. In comparison to perfusion of full human organs, a single vessel perfusion can be done with ~×100 less materials (e.g., ~25 mg of NP and 500 ml of perfusate in human kidneys). This dramatic reduction in materials reduces cost and makes replicates more experimentally tractable. In addition to less materials for the perfusion, the amount of relevant tissue is also significantly less. This makes it possible to have a full quantitative accounting of all materials and cells in the system. For example, essentially all ECs can be harvested and assessed, and off‐target sinks can be eliminated. This simplified system then allows for clear isolation of individual parameters of relevance to effective vascular targeting.

Third, human umbilical chord and placental tissues should be easily and frequently accessible to most researchers at a relatively low cost. Discarded umbilical cords and placenta are significantly more available than human organs for research. Additionally, several vascular grafts can be procured from the same donor and can be used in both control and experimental groups under identical experimental conditions. As an example, one umbilical cord can typically provide 6–10 identical (7–10 cm) vascular grafts. These features make evaluation of biologic replicates more feasible and thereby enable internal controls to account for donor to donor variability.

Finally, the design of the IVPS can be adapted for use with a variety of vessel diameters and lengths. In this study, we focused on showing quantitative effects with umbilical cord and placental arteries, but human coronary arteries and vessels from discarded human organs could be perfused as well. Large vessels from different often‐used animal models could also be used to evaluate species as a variable and thereby directly assess translational relevance. Additionally, the use of vessels containing vasa vasorum (either animal or human) could allow for assessment of the impact of the expanded surface area and altered hydrodynamics in this microcirculation relative to the large vessel.

In addition to these benefits of the IVPS, it is also essential to note the limitations of this system to ensure it is used in the proper context. First, the IVPS does not allow for assessment of NP targeting to microvascular beds as is possible in human organ perfusion or in vivo animal models. Moreover, complex capillary structures like those present in the renal glomerulus will undoubtedly present challenges different to those of an isolated single vessel. Nevertheless, we believe the IVPS can provide a complementary bridge between standard cell culture approaches and ex vivo organ perfusion. In this capacity, the IVPS has the potential to enable better translation of endothelial‐targeted nanomedicines.

## 4 CONCLUSION

We have built and tested an ex vivo isolated vessel perfusion system which provides a novel tool for quantitative testing of vascular‐targeted NPs. In comparison with standard static cell culture, which alters ECs properties, our system employs readily obtained vascular grafts, which are 3D structures with intact native vascular endothelial lining. The IVPS is a reliable testing system that is inexpensive and easy to produce. It can be adapted to test a variety of relevant targeting parameters, such as vessel size, type, pressure, flow rate, and shear stress. The system allows for the simultaneous perfusion of up to eight vascular grafts.

The IVPS has the potential to improve clinical translation of endothelial‐targeted nanomedicines by providing a native vascular context that retains the capacity for robust quantification without sacrificing translational relevance. Moreover, this ex vivo approach can facilitate subsequent in vivo experiments by treating human vessels prior to implantation in humanized mouse models.

## 5 MATERIALS AND METHODS

### 5.1 Materials

Poly (lactic acid)‐poly (ethylene glycol) (PLA–PEG) copolymer (Mw 16:5 kDa) was purchased from PolySciTech (West Lafayette, IN) and used without further purification. Anhydrous dimethyl sulfoxide (DMSO) was obtained from Sigma‐Aldrich (St. Louis, MO). 1,1′‐dioctadecyl‐3,3,3′,3′‐tetramethylindocarbocyanine perchlorate (DiI), 1,1′‐dioctadecyl‐3,3,3′,3′‐tetramethylindodicarbocyanine, 4‐chlorobenzenesulfonate salt (DiD), and 3,3′‐dioctadecyloxacarbocyanine perchlorate (DiO) fluorescent dyes were obtained from Thermo‐Fisher Scientific (Waltham, MA). *Ulex europaeus* type I lectin (UEA I) with or without fluorescein isothiocyanate and antifade mounting medium with 2‐(4‐amidinophenyl)‐6‐indolecarbamidine dihydrochloride, 4′,6‐diamidino‐2‐phenylindole dihydrochloride (DAPI) were acquired from Vector Laboratories (Burlingame, CA). Anti‐CD31/human platelet EC adhesion molecule‐1 (CD31/PECAM‐1) antibodies were obtained from Invitrogen (Carlsbad, CA). Anti‐ICAM‐2 antibodies were obtained from Biolegend (San Diego, CA). Antivascular endothelial cadherin (VE‐cadherin) monoclonal (clone 16B1) antibody was purchased from eBioscience and corresponding Goat anti‐Mouse IgG (H + L) Cross‐Adsorbed Secondary Antibody was purchased from Invitrogen (Carlsbad, CA). Triton X‐100 (t‐phenoxy polyethoxyethanol) was obtained from AmericanBio, Inc. (Canton, MA) and DAPI (4′,6‐diamidino‐2‐phenylindole, dihydrochloride; cat. No: 62247) were obtained from Thermo Scientific™(Waltham, MA). Fisherbrand Superfrost microscope slides and 22 × 40 mm cover slips were purchased from Fisher Scientific (Pittsburgh, PA) Ringer's lactate (LR) solution was obtained from Hospira (Lake Forest, IL) and Dulbecco's modified eagle's medium (DMEM), fetal bovine serum (FBS), l‐glutamine, and Penn/Strep were obtained from Gibco.

### 5.2 NP formulation

NPs were formulated following a well‐established nanoprecipitation method.^31^, ^34^ PLA–PEG copolymer was dissolved in DMSO to 50 mg/ml and mixed with lipophilic fluorescent dyes (DiI, DiO, and DiD) in a dye: polymer weight ratio of 0.5%, and then added dropwise into distilled water under vigorous agitation. This resulted in uniform NPs with a size of ~160 nm with a polydispersity index <0.2 which was confirmed with dynamic light scatter (DLS) measurements. Polymer NPs were then conjugated to targeting antibodies (Abs) or Ulex in excess ratio of 50 μg protein to 1 mg of polymeric NPs using 1‐ethyl‐3‐(3‐dimethylaminopropyl) carbodiimide hydrochloride mediated COOH─NH_2_ coupling under strong agitation for 1 hr. Successful conjugation of the antibody or protein was verified using a cell‐based binding assay previously described.^31^ In NP targeting experiments described below, NP background binding was assessed with either nonconjugated NP (control for Ulex‐NP) or Isotype‐conjugated NP (IgG2a control for ICAM2‐NP). Nonspecific binding was assessed quantitatively by flow cytometry and qualitatively by confocal microscopy (described below).

### 5.3 Perfusion system design

An ex vivo human vessel perfusion system was designed to mimic the physiological environment of blood vessels with easily manipulated perfusion parameters. A Masterflex L/S peristaltic pump with eight Masterflex L/S Easy‐Load pump heads (Vernon Hills, IL) was used to simultaneously pump up to eight closed perfusion loops with custom designed vessel perfusion chambers (see Figure 1a). Chemical resistant polyvinyl chloride (PVC) chambers were used to host a single isolated vascular graft for perfusion (2–12 cm in length). The vessels were cannulated and secured to needles (add the two sizes we mostly used) with 2–0 silk ties, and then mounted within the perfusion champers. Masterflex BioPharm Plus platinum silicone tubing and Cole‐Parmer luer fitting (Vernon Hills) were used to attach to the chamber and create a close perfusion loop system.

### 5.4 Human umbilical/placental artery procurement

De‐deidentified human umbilical cords (20–40 cm in length) with or without attached placentas were obtained from Yale‐New Haven Children's Hospital (New Haven, CT). To decrease damage and preserve the tissue, umbilical cords and placentas were transported, stored and dissected on ice (at 4°C) all times unless otherwise noted. To dissect umbilical vessels, the umbilical cords were fixed to dissection table with hypodermic needles and umbilical arteries were carefully dissected from surrounding connective tissue with sterile Metzenbaum scissors and DeBakey/atraumatic forceps. Intact arteries were cannulated and flushed with cold (4°C) LR solution to remove blood from the vessels and then cut into 10–12 cm segments in length. Isolated vessels were temporarily stored on ice in conical tubes filled with cold LR solution until all vessels were prepared for the experiment. To dissect placenta vessels, the umbilical cord was first cut off 1.5–2.0 cm from placentas. The remaining umbilical arteries were cannulated to flush the placenta with cold LR solution. Then placental arteries were gently dissected from the surface of the placentas. The selection of arteries for perfusion was based on diameter of the vascular graft required (for example, the internal diameter of vascular graft for mice surgery was 0.2–0.4 mm). The dissected vessels were stored in ice‐cold LR solution until being mounted in the perfusion chamber or cut into 0.5 cm segments for preperfusion staining (control).

We have found that tissue storage conditions have a pronounced effect on the quality of the endothelial layer prior to experimentation. As the tissue waits between the collection and the experiment, the endothelial layer becomes less continuous, with gaps in CD31+ cells on the luminal surface of the vessel. This progressive decline in endothelial quality is exacerbated as storage temperature increases from 4 to 18°C and to 37°C. Representative images of umbilical arteries stored under each of these conditions are shown in Figure S1. Based on these results, only blood vessels collected immediately after a C‐section were used for studies, to reduce variability in wait time and thus variability in the starting endothelium quality.

The perfusion system and the perfusion chambers were easily adapted to various vessel diameters and graft lengths by selecting the cannulated gavage needle diameter and the length of the perfusion chamber (see Figure 1b,c).

### 5.5 Characterizing flow profile

Based on the capabilities of the peristaltic pump and the diameter of the perfused vessel, a table of obtainable flow rates and resulting shear stress was calculated. Internal pressure at different flow rates with a constant tubing diameter was measured using a probe. The shear stress was calculated by the Hagen–Poiseuille formula:

(1) $$\tau =\frac{4\;\mathrm{Q\mu}}{\pi r^{3}}$$

where *τ*, wall shear stress; *Q*, volume flow rate; *μ*, viscosity of fluid (assumed at 0.01 dyne·s/cm^2^); and *r*, inner radius of cylindrical tube.^35^

These results are reported in Table 1.

### 5.6 EC isolation and flow cytometric analysis

For gentle EC isolation, vascular grafts were first washed with warmed PBS. The vessel was then filled with ~0.5 ml of a 0.1% solution of collagenase II in PBS (Worthington Biochemical, Lakewood, NJ) and incubated for 10 min at 37°C. The vessel was then flushed with approximately 1 ml of PBS + 1% BSA, and the flow through was collected in a microcentrifuge tube. Cells were collected from this suspension by centrifugation for 5 min at 3,500*g*, and then stained using fluorescently labeled antihuman PECAM‐1/CD31 (clone wm59, 5μg/ml) or an isotype control in PBS + 1% BSA for 50 min. Cells were then washed with PBS + 1% BSA and filtered for cytometric analysis. Cytometric light filters are selected to capture the EC stain (CD31) as well as fluorescence from bound NPs.

### 5.7 Whole‐mount *en face* immunofluorescence confocal microscopy

Whole‐mount *en face* immunostaining and confocal microscopy were used for visualization and evaluation of vascular endothelial layer and NP accumulation and retention. Pre‐ and postperfusion samples were collected by taking a 2 mm segment of the intact vessel and staining ECs with fluorescently labeled antihuman PECAM‐1/CD31 (clone wm59, 5 μg/ml) for 60 min at 4°C. Vessel segments were then washed twice with cold PBS + 1%BSA for 10 min prior staining nuclei with Hoescht (1 μg/ml PBS + 1% BSA) for 10 min. For VE‐Cadherin staining, vascular segment samples were fixed with 3% paraformaldehyde for 5 min and permeabilized with 0.5% Triton X‐100 in PBS for 5 min at room temperature. The samples were then washed with 1% PBS and incubated with antiVE‐cadherin (clone 16B1, 5 μg/ml) for 16 hr at 4°C. Samples were then washed and incubated for 60 min with fluorescently labeled secondary antibody (Goat anitmouse IgG, 10 μg/ml) incubation at 4°C. To remove unbound antibody the samples were washed three times in PBS. To stain cells nuclei the samples were incubated with DAPI nuclear stain (1 μg/ml)) for 10 min at 4°C. The samples were placed endothelial‐side up on microscope slides, coated with a drop pure glycerin (glycerol), and then cover slipped. To create as flat a surface as possible, silicone chemical resistant lubricant (Dow Corning, Midland, MI) was applied in a perimeter around the edge of the vessel segment to hold the cover slip in place. Fluorescent images were captured with an LSM 410 spinning‐disc confocal microscope and processed using Zen software (Carl Zeiss, Inc, Thornwood, NY). EC layer and junctional integrity were assessed with five randomly taken confocal images at ×10, ×40, and ×100 magnification per each sample.

### 5.8 Vascular grafts transplantation

All animal experiments were conducted in accordance with Yale University Animal Care and Use Committee guidelines (Protocol # 2018‐07863). Six CB17 severe combined immunodeficiency disease (SCID)/beige mice (*Mus Musculis*) (Taconic, Hudson, NY) were used for allograft implantation experiment at ages 5–8 weeks. Prior to surgery, the mice were anesthetized with an IP injection of ketamine (100 mg/kg) and xylazine (10 mg/kg). A midline laparotomy was used to expose infrarenal abdominal segment of the mouse abdominal aorta. Experimental vessel segments of 3–5 mm in length and of 1 mm inner diameter (approximating the caliber of murine aortae) were surgically implanted as end‐to‐end interposition grafts. The microsurgical anastomoses were performed using ×15–×18 magnification and 10–0 monofilament nylon suture (AROSurgical, Newport Beach, CA). Following completion of the anastomosis, vascular integrity was restored, hemostasis was assured, and graft blood flow was confirmed. Abdominal cavity was flushed with warmed sterile saline before closure. In the postoperative period animals were monitored and heating lights were used to avoid hypothermia. Animals recovered quickly with normal limb function, which was a reliable indicator of good graft patency and limb perfusion.

### 5.9 Implanted grafts histology and immunostaining

The implanted vascular grafts were explanted 7 days after implantation and then snap‐frozen. NP retention and endothelial preservation were evaluated using frozen sections, sliced longitudinally with 15 μm thickness, and stained using fluorescent antihuman CD31 as described above. Sections were mounted using antifade mounting medium with DAPI, and imaged using an EVOS fluorescent imaging system microscope.

### 5.10 Statistical analyses

The results of the experiments are expressed as the means ± the standard deviation. Statistical analyses were performed by ANOVA and a post hoc Tukey's test where appropriate for comparison between groups using Prism 8.0 (GraphPad Software, Inc.; La Jolla, CA). A value of *p* < .05 was considered statistically significant.

## CONFLICT OF INTEREST

The authors have no conflict of interest to declare.

## Supporting information


**Figure S1** *En face* confocal images of vessel segments perfused with NPs at 37°C for 1 hr, which are either immediately transferred to ice (a–c) or stored at room temperature for 2 hrs (d–f). Vessel segments are stained with anti‐CD31‐FITC (green) and Hoechst nuclear stain (blue); NPs are depicted in red. Scale bars are 20 μm.
**Figure S2.** Bar graphs depicting the percent of CD31+ endothelial cells harvested following perfusion as a function of flow rate (a) and representative confocal images of vessel segments from vessels perfused at 5 ml/min (b) and 2.5 ml/min (c). *En face* confocal images of vessel segments perfused with NPs at 1.5 ml/min (d), 2.5 ml/min (e), and 5 ml/min (f) which are stained with anti‐CD31‐FITC (green) and Hoechst nuclear stain (blue); NPs are depicted in red. Scale bars are 20 μm.
