# Supplementary material for: Ex vivo isolated human vessel perfusion system for the design and assessment of nanomedicines targeted to the endothelium
Source: Bioeng Transl Med. 2020 Jan 28;5(2):e10154. doi: 10.1002/btm2.10154 (PMC7237142; doi:10.1002/btm2.10154)
Supplement: Supplementary file 1 — Figure S1 En face confocal images of vessel segments perfused with NPs at 37°C for 1 hr, which are either immediately transferred to ice (a–c) or stored at room temperature for 2 hrs (d–f). Vessel segments are stained with anti‐CD31‐FITC (green) and Hoechst nuclear stain (blue); NPs are depicted in red. Scale bars are 20 μm. Figure S2. Bar graphs depicting the percent of CD31+ endothelial cells harvested following perfusion as a function of flow rate (a) and representative confocal images of vessel segments from vessels perfused at 5 ml/min (b) and 2.5 ml/min (c). En face confocal images of vessel segments perfused with NPs at 1.5 ml/min (d), 2.5 ml/min (e), and 5 ml/min (f) which are stained with anti‐CD31‐FITC (green) and Hoechst nuclear stain (blue); NPs are depicted in red. Scale bars are 20 μm. [file BTM2-5-e10154-s001.docx]

**
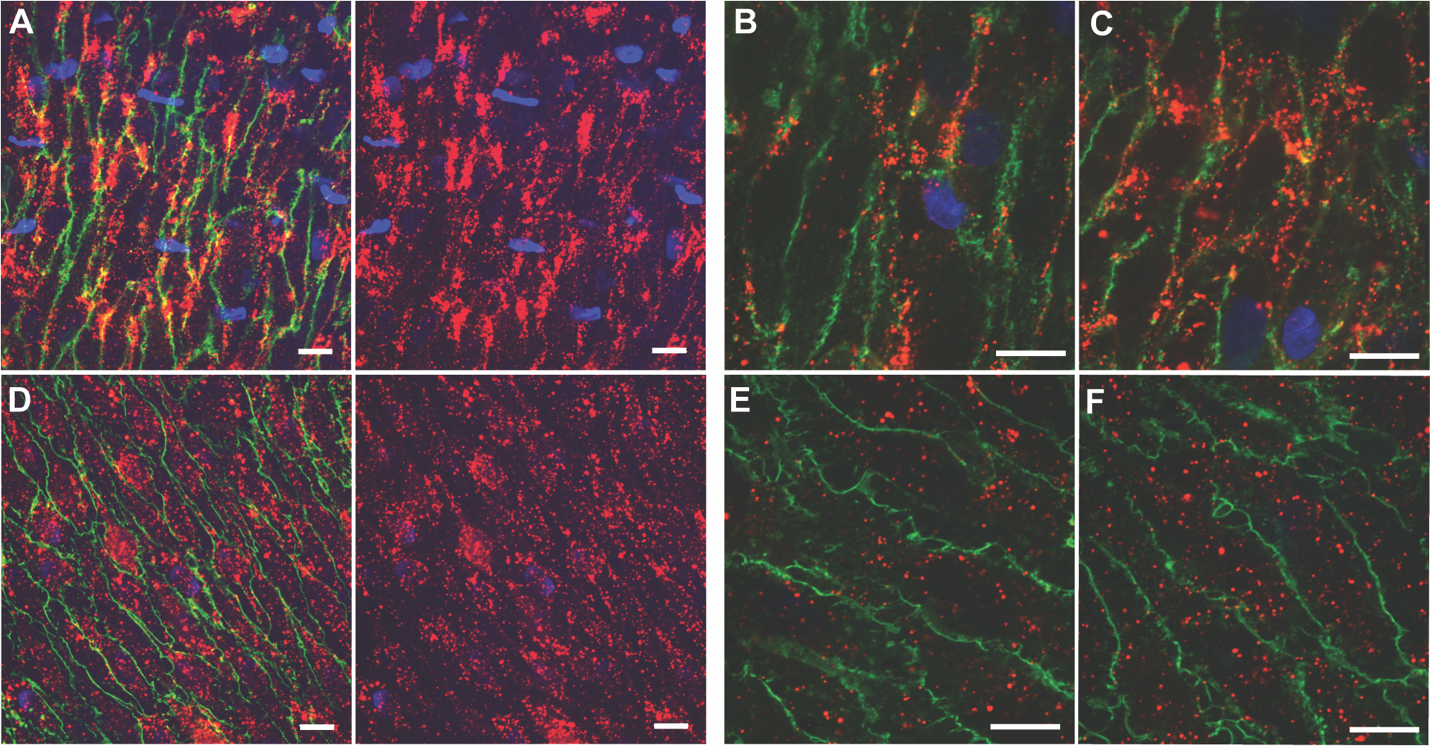
**

**Supplemental Figure 1.** *En face* confocal images of vessel segments perfused with NPs at 37C for 1 hour, which are either immediately transferred to ice (**A-C**) or stored at room temperature for 2 hours (**D-F**). Vessel segments are stained with antiCD31-FITC (green) and Hoechst nuclear stain (blue); NP are depicted in red. Scale bars are 20 µm.

**
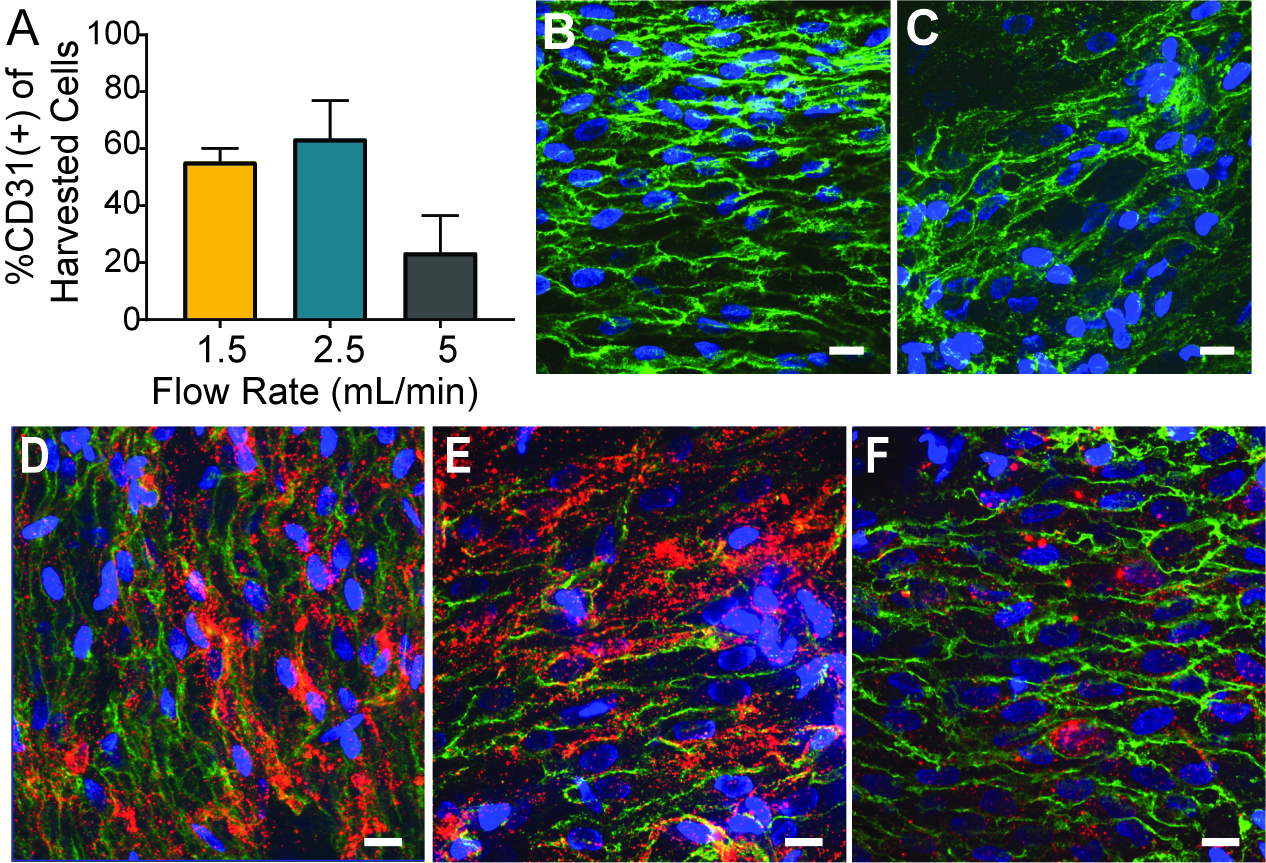
**

**Supplemental Figure 2.** Bar graphs depicting the percent of CD31+ endothelial cells harvested following perfusion as a function of flow rate (**A**) and representative confocal images of vessel segments from vessels perfused at 5mL/min (**B**) and 2.5mL/min (**C**). *En face* confocal images of vessel segments perfused with NPs at 1.5 mL/min (**D),** 2.5 mL/min (**E**)**,** and 5 mL/min (**F**) which are stained with antiCD31-FITC (green) and Hoechst nuclear stain (blue); NP are depicted in red. Scale bars are 20µm.
